# Gene-environment interaction between lead and Apolipoprotein E4 causes cognitive behavior deficits in mice

**DOI:** 10.1186/s13024-017-0155-2

**Published:** 2017-02-07

**Authors:** Anna K. Engstrom, Jessica M. Snyder, Nobuyo Maeda, Zhengui Xia

**Affiliations:** 1 Toxicology Program, Department of Environmental and Occupational Health Sciences, University of Washington, Box 357234, Seattle, WA 98195 USA; 2 Department of Comparative Medicine, School of Medicine, University of Washington, Seattle, WA 98195 USA; 3 Department of Pathology and Laboratory Medicine, University of North Carolina, Chapel Hill, NC 27599 USA

**Keywords:** Lead, Apolipoprotein E, Cognitive behavior, Adult hippocampal neurogenesis, Learning and memory

## Abstract

**Background:**

Alzheimer’s disease (AD) is characterized by progressive cognitive decline and memory loss. Environmental factors and gene-environment interactions (GXE) may increase AD risk, accelerate cognitive decline, and impair learning and memory. However, there is currently little direct evidence supporting this hypothesis.

**Methods:**

In this study, we assessed for a GXE between lead and ApoE4 on cognitive behavior using transgenic knock-in (KI) mice that express the human Apolipoprotein E4 allele (ApoE4-KI) or Apolipoprotein E3 allele (ApoE3-KI). We exposed 8-week-old male and female ApoE3-KI and ApoE4-KI mice to 0.2% lead acetate via drinking water for 12 weeks and assessed for cognitive behavior deficits during and after the lead exposure. In addition, we exposed a second (cellular) cohort of animals to lead and assessed for changes in adult hippocampal neurogenesis as a potential underlying mechanism for lead-induced learning and memory deficits.

**Results:**

In the behavior cohort, we found that lead reduced contextual fear memory in all animals; however, this decrease was greatest and statistically significant only in lead-treated ApoE4-KI females. Similarly, only lead-treated ApoE4-KI females exhibited a significant decrease in spontaneous alternation in the T-maze. Furthermore, all lead-treated animals developed persistent spatial working memory deficits in the novel object location test, and this deficit manifested earlier in ApoE4-KI mice, with female ApoE4-KI mice exhibiting the earliest deficit onset. In the cellular cohort, we observed that the maturation, differentiation, and dendritic development of adult-born neurons in the hippocampus was selectively impaired in lead-treated female ApoE4-KI mice.

**Conclusions:**

These data suggest that GXE between ApoE4 and lead exposure may contribute to cognitive impairment and that impaired adult hippocampal neurogenesis may contribute to these deficits in cognitive behavior. Together, these data suggest a role for GXE and sex differences in AD risk.

## Background

Alzheimer’s disease (AD) is the most common age-related neurodegenerative disorder [1]. Autosomal dominant mutations account for less than 5% of all Alzheimer’s cases [2], thus, most AD cases are likely due to a combination of environmental and genetic risk factors [3–5]. A gene-environment interaction (GXE) between genetic risk factors and environmental exposures may perturb hippocampus-dependent learning and memory, accelerate cognitive decline, and contribute to neurodegenerative diseases, including AD. However, there is a paucity of information supporting this GXE hypothesis.

Apolipoprotein E4 allele (ApoE4) is the strongest genetic risk factor for late-onset AD [6]; it increases the frequency and decreases the age at onset of AD in a gene dose-dependent manner [6]. ApoE4 carriers also have a worse prognosis following traumatic brain injury, a higher prevalence of mild cognitive impairment, and accelerated cognitive decline [7–11].

ApoE4 impairs hippocampus-dependent learning and memory in an age- and sex-dependent manner in transgenic knock-in (KI) mouse models [12–14]. In contrast to aging ApoE3-KI mice or male ApoE4-KI mice, female transgenic ApoE4-KI mice exhibit spatial learning and memory deficits starting at 16 months of age [12, 14]. Interestingly, female human ApoE4 carriers have an increased risk of AD compared to males, and female ApoE4 carriers with mild cognitive impairment experience greater hippocampal volume loss and reduced memory performance [15, 16]. Due to the negative effects of ApoE4 on cognition, ApoE4 carriers, particularly female carriers, may be more susceptible to toxicant-induced cognitive impairment compared to ApoE4 non-carriers.

Lead is a ubiquitous environmental contaminant and a significant public health concern in the U.S. and globally [17–19]. This is exemplified by the recent water crisis in Flint, Michigan, where the water supply was contaminated with lead due to lead leaching from old water pipes. The fact that many U.S. cities still have lead service pipes for water distribution underscores the continuing risk of lead exposure in the U.S. [20].

Both animal and epidemiological studies have reported an association between lead exposure and accelerated cognitive decline and/or AD-associated neuropathology in adults [21–25]. Interestingly, among workers occupationally exposed to lead, those with at least one ApoE4 allele experienced accelerated cognitive decline relative to ApoE4 non-carriers [26]. This epidemiological finding suggests that an interaction between lead and ApoE4 may accelerate cognitive decline. However, an experimental model is necessary in order to directly assess for a GXE between ApoE4 and lead regarding cognitive impairment. In this study, we used homozygous male and female ApoE3-KI and ApoE4-KI mice as an experimental model in order to assess the effect of lead exposure and ApoE4 on cognitive behavior and to examine any potential sex differences in susceptibility. We used deficits in hippocampus-dependent learning and memory as a measure of cognitive decline and AD-related memory impairment because the hippocampus is one of the earliest brain regions affected in AD patients and deficits in hippocampus-dependent spatial learning and memory may develop prior to the onset of a clinical diagnosis [27–29].

Interestingly, perturbation of adult hippocampal neurogenesis may cause cognitive behavior deficits, accelerate cognitive decline, and increase AD risk [30]. During adult hippocampal neurogenesis, adult neural precursor cells in the dentate gyrus (DG) of the hippocampus continuously generate adult-born neurons [31] which contribute to hippocampus-dependent learning and memory [32–37]. Various factors have been shown to modulate adult neurogenesis [31, 33, 34, 37, 38], however, little is known about whether toxicants or GXE may impair adult hippocampal neurogenesis. ApoE is expressed in adult neural precursor cells in the DG [6, 31, 39], and in vivo studies using ApoE4-KI mice found that ApoE4 alters adult-born neuron survival and maturation in an age- and sex-dependent fashion [12–14, 40]. Furthermore, we previously reported that lead can impair the proliferation, survival, and differentiation of adult neural precursor cells from the DG of the hippocampus in vitro [41]. No study to date has assessed the effect of adult-only lead exposure on adult hippocampal neurogenesis and cognitive behavior. Thus, we also investigated whether impaired adult hippocampal neurogenesis may contribute to this GXE between ApoE4 and lead exposure regarding cognitive impairment.

## Methods

### Mice

Humanized Apolipoprotein E3 and E4 knock-in (ApoE3-KI and ApoE4-KI) mice were generated as previously described [42] and provided by Dr. Nobuyo Maeda at the University of North Carolina, Chapel Hill. The human E3 or E4 allele in the ApoE3-KI or ApoE4-KI mice is expressed at physiological levels under control of the endogenous mouse ApoE promoter [13]. ApoE3-KI and ApoE4-KI animals were maintained as homozygous lines and all animals were housed in standard conditions (12 h light/dark cycle) with food and water provided ad libitum.

### Reagents

Animal drinking water with 0.2% lead (II) acetate (Cat. 316512, Sigma-Aldrich, St. Louis, MO) was prepared from a stock lead acetate solution and replaced weekly. The preparation, use, and disposal of hazardous agents was carried out according to the Environmental Health and Safety Office at the University of Washington.

### Lead exposure

Male and female ApoE3-KI and ApoE4-KI animals were weaned at 28 days and littermates of the same sex were randomly mixed into groups of 3–5 animals per cage. At 8 weeks of age, the mice were either switched to drinking water with 0.2% lead acetate (lead-treated) or kept on normal animal drinking water (control). Body weight was recorded every 1–2 weeks throughout the exposure. Water consumption was monitored for the first week of the exposure period. Lead-treated animals were exposed to lead drinking water for 12 weeks, at which point all the animals were switched to normal drinking water for the remaining duration of the study for the behavior cohort. We used separate cohorts of animals, one for behavior and one for cellular studies, to assess cognitive behavior and adult hippocampal neurogenesis, respectively. The behavior cohorts had *n* = 8–13 animals per genotype/treatment/sex and the cellular cohorts had *n* = 6–10 animals per genotype/treatment/sex.

### Open field test

The open field test was conducted before and after the lead exposure to assess for the development of any lead-induced locomotor deficits or anxiety. Briefly, each animal was placed into a TruScan Photo Beam Tracking arena (Colbourn Instruments, Whitehall, PA) with Plexiglas walls (10″ X 10″ X 16″) and their movement was monitored with two sets of infrared breams. The animal was allowed to freely explore the arena for 20 min and the data was collected by TruScan 2.0 software (Colbourn Instruments).

### Elevated plus maze

We conducted the elevated plus maze test in order to assess the effect of lead exposure on anxiety. The maze (26″ x 26″ x 15.25″; San Diego Instruments, San Diego, CA) consisted of four plastic arms with 7″ walls (two enclosed and two open), and it was placed in the center of the behavior room. Each animal was placed into the center of the maze facing an open arm and allowed to freely explore the maze for 5 min. The open and closed arm ends were defined as the distal 1/3 of the arms. ANYmaze software (San Diego Instruments) was used to collect data on the animal’s movement during the test.

### Novel object location test

In order to assess hippocampus-dependent, short-term spatial working memory, we used the 1 h novel object location (NOL) test. This test was performed as previously described with a few modifications [34]. Briefly, each animal was placed into an open field arena (Colbourn Instruments) with two identical objects in two different locations. During training, the animal was allowed to freely explore the two objects for 5 min and then returned to its home cage. After 1 h, the animal was returned to the arena with the same two objects; one object remained in its original location and one object had been moved to a novel location. The time the animal spent actively investigating each object during the training and testing was quantified. Each training and testing session was recorded and scored offline by an experimenter blinded to the animal’s genotype and treatment. We calculated the discrimination index by dividing the difference in exploration time between the novel (C) and familiar (A) locations by the total exploration time.

### Cued-contextual fear-conditioning

Contextual fear conditioning is another form of hippocampus-dependent learning and memory [33, 37, 43]. We tested 5–6-month-old animals and used a weak foot shock conditioning paradigm (3 x 0.3 mA, 2 s shocks with 2 min inter-trial intervals) as previously described [33]. For conditioning, the mouse was placed into the foot shock context (10″ x 10″ x 16″ arena with grid shock floor (Colbourn Instruments) and star-shaped wallpaper) and allowed to freely explore the arena for 2 min before the presentation of a 90 dB, 30 s tone (conditioned stimulus, CS). During the last 2 s of the tone presentation, a 0.3 mA foot shock (unconditioned stimulus, US) was delivered via the grid shock floor. This cycle was repeated two more times for a total of three cycles before the animal was returned to its home cage. The CS and US were automated and delivered by TruScan software (Colbourn Instruments). The contextual fear memory test was conducted 24 h after conditioning. The mouse was placed back into the foot shock context for 2 min in the absence of any foot shock. For the cued test (performed 2 h after context test), the animal was placed into a different context (new room; hexagonal Plexiglas arena; cartoon wallpaper) and allowed to freely explore for 2 min followed by the presentation of the CS for 2 min. For the novel context test (performed 2 h after the cued test), the mouse was placed into a novel context (new room; rat cage; striped wallpaper) and allowed to freely explore without any CS or US presentation. In all three tests, persistent freezing behavior (four paws on the ground, no head or body movement besides breathing) was recorded manually every 5 s during the 2 min scoring periods by an experimenter blinded to animal genotype and treatment.

### T-maze continuous alternation task

We assessed spontaneous alternation in 12–13- month-old animals using a continuous alternation T-maze protocol with slight modifications [44]. Briefly, the black, plastic T-maze had two goal arms and a start arm (12.2″ x 4.5″ x 8.26″), and was placed on a platform (22.5″) in the center of a room. The test consisted of a first-forced trial followed by 14 free-choice trials. For the first-forced trial, one of the goal arms was (randomly) blocked with a plastic guillotine door. The animal was sequestered in the distal one-third of the start arm for 5 s before the guillotine door was raised and the animal was allowed to enter the unblocked goal arm. Once the animal returned to the start arm, it was sequestered in the start arm for 5 s before the start of the 14 free-choice trials. For each free-choice trial, the mouse was allowed to enter either of the unblocked goal arms; once it entered a goal arm, the other goal arm was blocked with a guillotine door. The animal eventually returned to the start arm and was sequestered for 5 s while all of the goal arms were unblocked. This was repeated for a total of 14 free-choice trials. The alternation percentage was calculated by dividing the number of times the animal entered alternating arms by 14 (free-choice trials). We defined arm entry as the animal’s tail tip entering the arm and defined repetitive arm entries as an animal re-entering the same arm three times in a row (e.g., 5 sequential entries into the same arm is 3 repetitive entries). An experimenter blinded to animal genotype and treatment scored each test.

### Brain and blood lead analysis

We collected blood and brain tissue for lead analysis at sacrifice after the cessation of the lead exposure from a subset of the mice in the cellular cohort (20 weeks old; *n* = 3–5 per genotype/sex/treatment). The Environmental Health Laboratory at the University of Washington measured blood lead levels using inductively coupled plasma mass spectrometry (ICP-MS; Agilent 7500ce, Agilent Technologies, Santa Clara, CA). The spatial distribution and semi-quantitative measurement of lead and zinc levels (ppm) in 30 μm brain hemisphere sections from 20-week-old ApoE4-KI control or lead-treated females was determined by laser ablation ICP-MS (LA-ICP-MS) using an NWR213 laser ablation system (ESI, Portland, OR) connected to an Agilent 7500ce ICP-MS (Agilent Technologies, Santa Clara, CA). Quantification was achieved using spiked protein matrix as external standards.

### TUNEL staining

TUNEL staining was performed using the Trevigen TACS 2 TdT-fluor In situ Apoptosis Detection Kit (Cat. 4812-30-K, Sigma-Aldrich, St. Louis, MO) on 30 μm fresh frozen coronal brain sections according to the manufacturer’s instructions. The cells were permeabilized using Trevigen’s Cytonin^TM^ permeabilization buffer and an experimental positive control was included (treated with TACS-Nuclease^TM^).

### Quantification of dentate gyrus volume

The volume of the dentate gyrus was quantified using coronal images (20X magnification) from 15-month-old female ApoE3-KI and ApoE4-KI mice from the behavior cohorts as previously described [45].

### BrdU administration

5-bromo-2′-deoxyuridine (BrdU) was from Sigma (Cat. B9285, Sigma-Aldrich, St. Louis, MO) and stored as a 20 mg/ml stock in saline with 0.007% NaOH at -20 °C. After 9 weeks of lead exposure, the mice in one of the cellular cohorts were dosed with 100 mg/kg BrdU by intraperitoneal injection 5 times in 1 day (every 2 h) and sacrificed 3 weeks later (at the end of the 12 week lead exposure) in order to assess the maturation of BrdU-labeled adult-born cells. A second cellular cohort was dosed with BrdU 1 X 100 mg/kg 2 h prior to sacrifice (at the end of the 12 week lead exposure period) in order to assess for proliferative BrdU-labeled cells.

### Immunohistochemistry

The primary antibodies and dilutions used in immunohistochemistry were: rat monoclonal anti-BrdU (1:500, Bio-Rad Laboratories AbD Serotec, Raleigh, NC), mouse monoclonal anti-NeuN (1:1000, Millipore, Billerica, MA), mouse monoclonal anti-GAD67 (1:2000, Millipore, Billerica, MA), and donkey polyclonal anti-DCX (1:200, Santa Cruz Biotechnology, Dallas, TX), Goat anti-rat, goat anti-mouse, and donkey anti-rat Alexa Fluor-conjugated secondary antibodies as well as Hoechst 33342 (2.5 μg/ml) were from Invitrogen (Carlsbad, CA). All of the primary and secondary antibodies were diluted into the appropriate blocking buffer (10% donkey or goat serum and 1% BSA).

Mice were anesthetized with ketamine/xylazine and then euthanized by decapitation. One brain hemisphere was freshly frozen for brain lead analysis or other biochemical analysis, while the other hemisphere was post-fixed in 10% neutral-buffered formalin for 3–4 days followed by 30% (w/v) sucrose in phosphate-buffered saline (PBS, pH 7.4) at 4 °C for 3–4 days until saturated. Each post-fixed hemisphere was then stored in cryoprotectant media (30% glycerol; 30% ethylene glycol; 40% PBS) at -80 °C until sectioning. Coronal brain sections (30 μm thick) were used for immunohistochemistry (IHC) using the free-floating antibody staining method as previously described [38].

### Quantification and imaging of immunostained cells

Immunostained cells were quantified as previously described [34, 38]. Every eighth serial section (30 μm) was immunostained for each IHC marker (or combination of markers). An experimenter blinded to treatment and genotype quantified marker^+^ cells in the subgranular zone and granule cell layer of the DG. This number was multiplied by 8 in order to estimate the total number of marker^+^ cells per DG. Marker colocalization (double-positive cell) was defined as overlapping fluorescent signals in a single cell using a Z-series stack. All the marker^+^ cells from at least 9 coronal sections per mouse (*n* = 3–5 mice per genotype/sex/treatment) were quantified for each immuno marker or marker combination. All images were captured with an Olympus Fluoview-1000 laser scanning confocal microscope with the following lenses: numerical aperture (NA) 0.75 10X, NA 0.75 20X, NA1.3 40X (oil), or NA 1.35 60X (oil). Optical Z-sections (1 μm thick) were collected and processed using ImageJ software (NIH, Bethesda, MD). Images were uniformly adjusted for color, brightness, and contrast with Adobe Photoshop CS4 (Adobe Systems Inc., San Jose, CA).

### Quantification of dendritic morphology

The dendritic morphology of DCX^+^ cells in control and lead-treated ApoE3-KI and ApoE4-KI mice (at least 13 individual neurons per genotype/sex/treatment) was assessed by Sholl analysis as previously described [34].

### Histology

Kidney and liver sections from control and lead-treated ApoE3-KI and ApoE4-KI males and females (*n* = 3–4 per genotype/treatment) were fixed in 10% neutral buffered formalin, paraffin embedded, cut into 4- to 5-μm sections, routinely stained with hematoxylin and eosin (H&E), and evaluated by a board-certified veterinary pathologist. Slides were coded to remove information regarding genotype and treatment. Kidneys were scored semi-quantitatively using a 0–4 scale, in which “0” was normal; “1” indicated minimal expansion of the glomerular mesangial matrix and minimal multifocal tubular protein; “2” indicated mild changes of the glomerulus with mild interstitial fibrosis, interstitial nephritis, and tubular protein; “3” indicated moderate multifocal to coalescing changes characterized by more pronounced glomerular, tubular and interstitial changes; and “4” indicated end stage disease with obsolescent glomeruli and severe multifocal to coalescing interstitial nephritis and fibrosis with or without dilation of the renal pelvis (hydronephrosis). Intranuclear inclusion bodies within the proximal renal tubules were noted when present. Livers were assessed using modified criteria from a previous study [46] and scored semi-quantitatively for changes including necrotic foci, cytoplasmic vacuolation, and mononuclear cell infiltration on a 0–4 system in which “0” was normal; “1” indicated minimal changes; “2” indicated mild changes; “3” indicated moderate changes; and “4” indicated severe changes. Images of representative lesions were acquired using NIS-Elements BR 3.2 64-bit and plated in Adobe Photoshop Elements. Image brightness and contrast was adjusted using Auto Smart Fix and Auto White Balance manipulations applied to the entire image.

### Statistical analysis

Statistical analyses were conducted using GraphPad Prism software (version 6.0 h for Mac, GraphPad Software Inc., San Diego, CA, USA) and Stata (version 12.0 for Mac, StataCorp LP, College Station, TX, USA). Two-way analysis of variance (ANOVA) with the Holm-Sidak *post-test* (α = 0.05) was used to analyze the open field, elevated plus maze, contextual fear, and T-maze data in order to account for the main effect of genotype (ApoE3-KI vs. ApoE4-KI), treatment (control vs. lead), or time. Two-tailed *t-*test (α = 0.05) was used for within-genotype comparisons for the NOL data. Multilevel mixed-effects linear regression (α = 0.05) was used for longitudinal analysis of within-genotype NOL discrimination index data. Two-way analysis of variance (ANOVA) with Fisher’s LSD *post-test* (α = 0.05) was used to analyze all of the cellular data. All data represent mean ± SEM, n.s. not significant; * *p* < 0.05; ** *p* < 0.01; *** *p* < 0.001.

## Results

### ApoE3-KI and ApoE4-KI mice do not exhibit weight loss, locomotor deficits, or anxiety following lead exposure

**Fig. 1:**
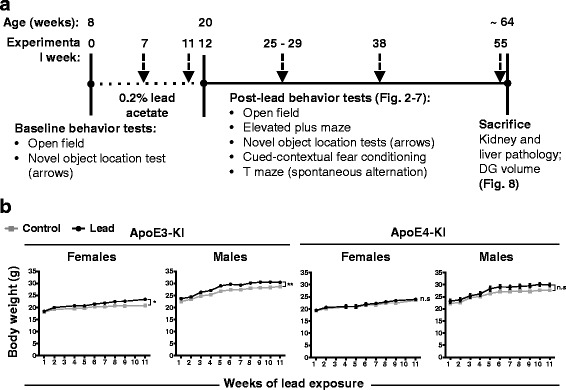
Adult male and female ApoE3-KI and ApoE4-KI mice do not exhibit weight loss upon exposure to 0.2% lead acetate for 12 weeks. **a** Experimental design and timeline for the behavior cohort. **b** Body weight was measured every 1–2 weeks during the lead exposure window. The lead-treated mice did not exhibit any weight loss relative to controls of the same sex and genotype. ApoE3-KI mice exposed to lead weighed slightly but significantly more at several time points than control mice during the lead exposure. Data are mean ± SEM with *n* = 8–13 per genotype/sex/treatment. Two-way ANOVA, significant main effect of treatment: n.s., not significant; * *p* < 0.05; ** *p* < 0.01

We exposed 8-week-old male and female ApoE3-KI and ApoE4-KI mice to 0.2% lead acetate (lead-treated) or normal drinking water (control) for 12 weeks to model sub-chronic environmental and occupational exposure through ingestion (Fig. 1). We used 0.2% lead acetate in our study because this concentration has been used extensively in the literature to study lead neurotoxicity in mice [47–52]. We recorded body weight every 1–2 weeks during the lead exposure period and did not observe any weight loss in any of the lead-exposed animals at any time during lead exposure (Fig. 1b). Lead-treated ApoE3-KI mice were slightly heavier than their controls at several time points, but this weight gain was not associated with any locomotor deficits. There was no significant difference in body weight between control and lead-treated ApoE4-KI mice.

We conducted the open field test for all of the animals in the behavior cohort before and after the 12-week lead exposure in order to assess locomotor activity and anxiety. The pre-exposure test was performed to exclude any intrinsic differences between the control and lead-treated cohorts that could complicate data interpretation. Although there were some small differences in locomotor activity between the two genotypes, with ApoE4-KI mice exhibiting slightly higher baseline activity than ApoE3-KI mice, there were no significant differences in open field activity between control and lead-treated cohorts prior to the lead exposure of either genotype (data not shown).

**Fig. 2:**
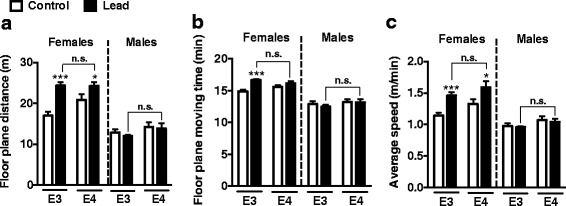
Lead-treated ApoE3-KI and ApoE4-KI male and female mice do not exhibit locomotor deficits in the open field test compared to controls. Mice were exposed to lead as previously described and the open field test was conducted after the cessation of the lead exposure. There was a significant main effect of lead treatment in female animals on floor plane distance, moving time, and average speed (Two-way ANOVA: floor plane distance, F(1,31) = 22.85, *p* < 0.0001; moving time, F(1,31) = 20.47, *p* < 0.0001; average speed, F(1,31) = 20.49, *p* < 0.0001). *Post-hoc* analyses found that lead-treated ApoE3-KI and/or ApoE4-KI females traveled a (**a**) greater distance, (**b**) spent the same or more time moving, and (**c**) traveled slightly faster than controls. There were no significant differences between lead and control ApoE3-KI and ApoE4-KI males or between lead-treated ApoE3-KI and ApoE4-KI mice (males or females) in any of the locomotor endpoints. Data are mean ± SEM with *n* = 8–13 per genotype/sex/treatment. Two-way ANOVA with Holm-Sidak *post-hoc* tests: n.s., not significant; * *p* < 0.05; *** *p* < 0.001

After lead exposure, the lead-treated females, both ApoE3-KI and ApoE4-KI, traveled further, spent more time moving, and moved slightly faster than their respective controls (Fig. 2). There was no significant effect of lead on locomotor activity in male mice. It is important to note that there were no significant differences in locomotor activity between lead-treated ApoE3-KI and ApoE4-KI mice of either sex.

**Fig. 3:**
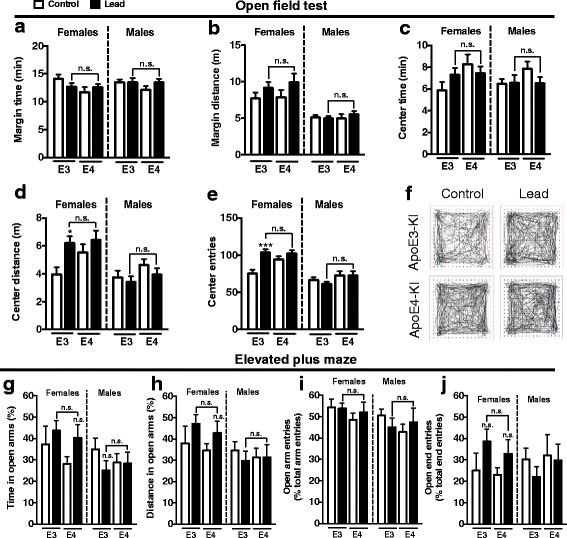
Male and female ApoE3-KI and ApoE4-KI mice exposed to lead do not exhibit overt anxiety in the open field test or elevated plus maze. The open field test was used to determine if lead treatment causes anxiety, measured as more time or distance in the margin, less time or distance in the center, or reduced center entries. There was a significant main effect of lead treatment in female mice on center distance and center entries (Two-way ANOVA: center distance, F_(1,31)_ = 5.936, *p* = 0.0208; center entries, F_(1,31)_ = 14.76, *p* = 0.0006). *Post-hoc* analyses found that there were no significant differences between lead-treated ApoE3-KI and ApoE4-KI mice (males and females) on the (**a**) time or (**b**) distance traveled in the margins, the (**c**) time or (**d**) distance traveled in the center, or the (**e**) number of center entries in the open field test. *D*, ApoE3-KI females treated with lead traveled a slightly greater distance in the center and *E*, made more center entries than controls. There were no significant differences between control and lead-treated ApoE4-KI females, ApoE3-KI males, or ApoE4-KI males in the open field test. **f** Representative open field track plots from female control and lead-treated ApoE3-KI and ApoE4-KI mice. In the elevated plus maze, both male and female ApoE3-KI and ApoE4-KI mice exposed to lead spent a (**g**) similar or greater amount of time and traveled a (**h**) similar or greater distance in the open arms of the maze compared to controls. Lead-treated males and females did not exhibit reduced (**i**) open arm or (**j**) open arm end entries compared to control animals. Data are mean ± SEM with *n* = 8–13 per genotype/treatment. Two-way ANOVA with Holm-Sidak *post-hoc* tests: n.s., not significant; * *p* < 0.05; *** *p* < 0.001

We also used the open field test to determine if lead treatment caused anxiety, measured as more time or distance in the margin, less time or distance in the center, or reduced center entries. Lead treatment did not change these parameters in males (Fig. 3a-f). Lead-treated ApoE3-KI females traveled a greater distance in the center of the open field and made more center entries compared to ApoE3-KI control females (Fig. 3d-f). Notably, there were no significant differences in center distance traveled and center entry between lead-treated ApoE3-KI and ApoE4-KI females.

We also conducted the elevated plus maze test to assess anxiety after the cessation of the lead exposure (Fig. 3g-j). The elevated plus maze is commonly used to assess the anxiogenic and anxiolytic effects of toxicological and pharmacological agents. It relies on a mouse’s natural avoidance of heights and open spaces and it’s preference for dark, sheltered spaces [53]. There was no significant effect of genotype or lead exposure on the percent of total time males or females spent or the total distance they traveled in the open arms by two-way ANOVA (Fig. 3g, h). In addition, there was no significant effect of genotype or treatment on the number of open arm entries or open arm end entries among males or females (Fig. 3i, j).

Thus, subchronic exposure of 8-week-old mice to 0.2% lead acetate did not decrease locomotor activity or cause anxiety in ApoE3-KI or ApoE4-KI mice. Although lead exposure may result in a slight hyperactive phenotype in ApoE3-KI and ApoE4-KI females and a slight anxiolytic phenotype in ApoE3-KI females, there were no significant differences between lead-treated ApoE3-KI and ApoE4-KI mice of either sex.

### ApoE4-KI females exposed to lead exhibit impaired contextual fear memory

**Fig. 4:**
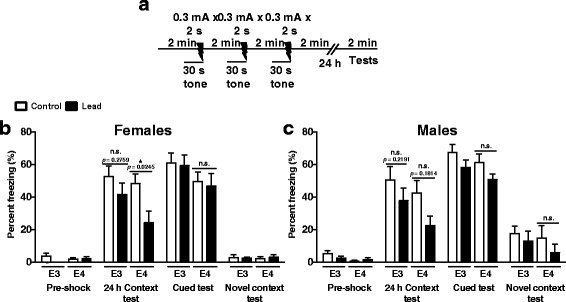
Lead impairs contextual fear memory in lead-treated ApoE4-KI females at 22–24 weeks of age. **a** Schematic of cued and contextual fear conditioning test performed post-lead exposure. **b**, **c** Female and male mice had low baseline freezing behavior (Pre-shock). In the 24 h Context test, there was a significant main effect of lead treatment on freezing behavior in both males and females (Two-way ANOVA: females, F_(1,33)_ = 6.803, *p* =0.0136; males, F_(1,42)_ = 4.435, *p* =0.0412). Although lead treatment reduced contextual memory in all animals (manifested as reduced freezing 24 h after fear conditioning) *post-hoc* tests revealed that this was only statistically significant between control and lead-treated ApoE4-KI females (Holm-Sidak *post-hoc* test: F_(1,33)_ = 2.648, *p* = 0.0245). Auditory-cued (hippocampus-independent) fear memory was not affected (Cued test) in any lead-exposed animals. All animals did not freeze when placed into a novel, non-shock context (Novel context test). Data are mean ± SEM with *n* = 8–13 per sex/genotype/treatment. Two-way ANOVA with Holm-Sidak *post-hoc* test. n.s. not significant; * *p* < 0.05

We also conducted a contextual fear-conditioning test (age of mice: 5–6 months old) to characterize the effect of lead and ApoE4 on hippocampus-dependent learning and memory (Fig. 4a). We used a 0.3 mA x 3 foot shock paradigm because we previously reported that this paradigm is sensitive to changes in contextual fear memory and adult neurogenesis [33]. All mice showed minimal freezing behavior at baseline before foot shock (Fig. 4b, c). We performed the context test 24 h after fear-conditioning in order to assess contextual fear memory. Interestingly, lead-treated ApoE4-KI females had significantly reduced contextual fear memory compared to ApoE4-KI controls (Fig. 4b). Male ApoE4-KI mice and both male and female ApoE3-KI mice exposed to lead exhibited a statistically non-significant reduction in freezing behavior upon lead treatment (Fig. 4b, c). Lead treatment did not cause any statistically significant changes in any animals in auditory-cued fear memory–a form of amygdala-dependent, but hippocampus-independent memory–or in general freezing in a novel context. Furthermore, lead treatment did not affect animals’ shock sensitivity (data not shown). Thus, lead exposure impairs the formation and/or retrieval of contextual fear memory in ApoE4-KI female mice.

### ApoE4-KI females exhibit decreased spontaneous alternation and increased repetitive arm entry in a T-maze

**Fig. 5:**
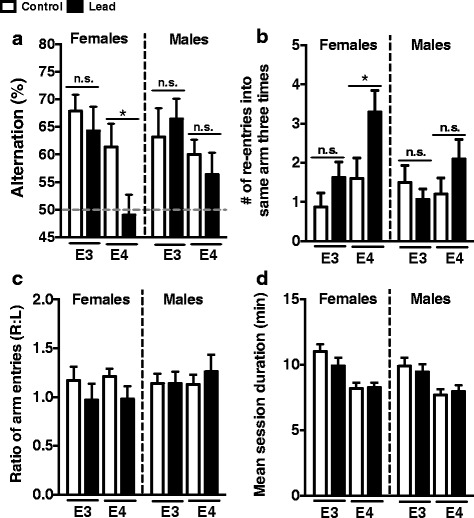
Lead impairs spontaneous alternation and increases repetitive arm entry in lead-treated ApoE4-KI females. Spontaneous alternation was assessed using the T-maze at 12–13 months of age. **a** There was a significant main effect of genotype on spontaneous alternation in females (Two-way ANOVA: genotype, F_(1,32)_ = 7.666, *p* = 0.0093). Lead-treated ApoE4-KI female mice exhibited reduced spontaneous alternation compared to female ApoE4-KI control mice (Holm-Sidak *post-test*: F_(1,32)_ =2.356, *p* =0.0490). **b** There was a significant main effect of both genotype and treatment on repetitive arm entries in females (Two-way ANOVA: genotype, F_(1,32)_ = 5.915, *p* =0.0208; treatment, F_(1,32)_ = 6.164, *p* =0.0185). ApoE4-KI females exposed to lead exhibited significantly increased repetitive arm entries compared to control ApoE4-KI female mice (Holm-Sidak *post-test:* F_(1,32)_ = 2.584, *p* 0.0289). There was no significant difference in spontaneous alternation or arm re-entries in lead-treated ApoE3-KI and ApoE4-KI male mice. **c** Female and male mice did not exhibit any arm preference. **d** There was a significant main effect of genotype on mean session duration in both the females and males, with ApoE4-KI mice completing the alternation task slightly faster than ApoE3-KI mice of the same sex (Two-way ANOVA: females, F_(1,30)_ = 19.50, *p* = 0.0001; males F_(1,41)_ =9.803, *p* = 0.0032). There was no significant difference in mean session duration between control and lead-treated animals of the same genotype and sex. Data are mean ± SEM with n = 8–13 per sex/genotype/treatment. n.s. not significant; * *p* < 0.05

We assessed spontaneous alternation using the T-maze in 12–13-month-old animals. Rodents spontaneously alternate the arms they enter in the T-maze, and this is likely due to a combination of novelty-elicited exploratory behavior as well as spatial working memory [44, 54]. Interestingly, lead-treated ApoE4-KI females exhibited a significant reduction in spontaneous alternation (Fig. 5a) and had a significant increase in repetitive arm entries compared to control ApoE4-KI females (Fig. 5b). These behavior changes were specific to females and were not observed in males. There were no significant differences between control and lead-treated animals on the ratio of left/right arm entries or mean session duration in either females or males (Fig. 5c, d). These data suggest that there may be a GXE interaction between lead and ApoE4 on spontaneous alternation and repetitive entries. Furthermore, lead-treated female ApoE4-KI mice are more sensitive than males. These differences are not due to a bias in arm entries or significant differences in task completion time.

### ApoE3-KI and ApoE4-KI mice exposed to lead exhibit a GXE interaction and sex differences in the onset of short-term spatial memory deficits in the novel objection location test (NOL)

We conducted a 1 h NOL test to assess hippocampus-dependent spatial working memory before, during, and after the lead exposure. Importantly, at each NOL time point (baseline through 10 months post-lead), none of the mice exhibited a preference for either object or location during the training session, and there was no difference in the total exploration time between lead-treated and control mice (data not shown).

**Fig. 6:**
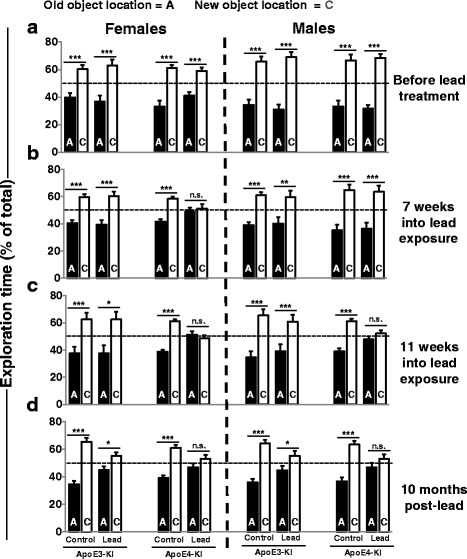
The effect of lead on short-term spatial memory in the NOL test. The NOL test was performed before**,** during, and after the lead exposure to assess for spatial working memory deficits. The time animals spent investigating the object in the old (location A) vs. new (location C) locations was quantified. More time spent exploring the object in the novel vs. old location indicates memory for the old location. **(** ***A*** **)** All the animals had intact spatial memory prior to the lead exposure. **(** ***B*** **)** At 7 weeks into the lead exposure, only the lead-treated ApoE4-KI females did not discriminate between the old vs. new object locations (Two-tailed *t*-test (A vs. C): *p* = 1.000). **(** ***C*** **)** At 11 weeks, both the lead-treated ApoE4-KI females and males no longer discriminated between the object locations (females, *p* = 0.4042; males, *p* = 0.1959) while the ApoE3-KI females and males spent significantly more time exploring the novel object location (females, *p* = 0.0107; males, *p* = 0.0098). **(** ***D*** **)** Lead-treated ApoE4-KI females and males continued to exhibit a deficit in spatial working memory at 10 months post-lead exposure (females, *p* = 0.0961; males, *p* = 0.1855). Lead-treated ApoE3-KI mice still spent statistically significantly more time exploring the novel object location at 10 months post-lead exposure (females, *p* = 0.0186; males, *p* = 0.0320). Data are mean ± SEM with *n* = 8–13 per genotype/treatment. Two-tailed *t*–test: n.s. not significant, * *p* < 0.05; ** *p* < 0.01; *** *p* < 0.001

Before lead exposure, all of the ApoE3-KI and ApoE4-KI females and males spent significantly more time exploring the object in the novel vs. familiar (C vs. A) location during the test period, indicating that they remembered the original object location (Fig. 6A). Seven weeks into the lead exposure, all of the mice spent significantly more time exploring the novel location except for lead-treated ApoE4-KI females, which did not discriminate between the old vs. new object locations (Fig. 6B). After 11 weeks of lead exposure, both the lead-treated ApoE4-KI females and males no longer discriminated between the familiar and novel object locations while the lead-treated ApoE3-KI mice continued to spend significantly more time exploring the novel object location (Fig. 6C). Both the lead-treated ApoE4-KI females and males continued to exhibit a deficit in spatial working memory at 3, 6, and 10 months post-lead exposure (Fig. 6D and data not shown). Lead-treated ApoE3-KI mice still spent significantly more time exploring the novel object location at 3, 6, and 10 months post-lead exposure (Fig. 6D and data not shown). However, the differences in exploration time between the new vs. old object locations became smaller over time among the ApoE3-KI mice exposed to lead indicating poorer spatial memory.

**Fig. 7:**
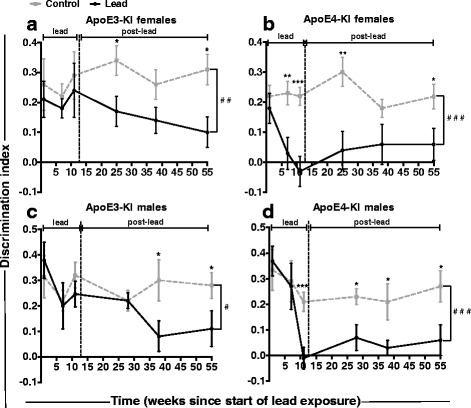
Genotype and sex differences in lead-induced reduction of discrimination index over time. The discrimination index in the NOL test was calculated by dividing the difference in exploration time between the novel (**c**) and familiar (**a**) locations by the total exploration time, and used to compare changes of spatial memory over time. There was a main effect of lead exposure on the discrimination index in all lead-exposed animals (Multiple mixed-effects linear regression: ApoE3-KI females, *p* = 0.001; ApoE4-KI females, *p* < 0.0001; ApoE3-KI males, *p* = 0.019; ApoE4-KI males, *p* < 0.0001). **a** There was no difference in the discrimination index between control and lead-treated ApoE3-KI females during the lead exposure (Two-tailed *t*-test: 7 week, *p* = 0.3046; 11 week, *p* = 0.9977). Lead-treated ApoE3-KI females had a significantly lower discrimination index at 3 and 10 months post-lead exposure (3 months, *p* = 0.0268; 10 months, *p* = 0.0156) and a non-significant decrease 6 months post-lead (*p* = 0.0901) compared to controls. **b** Lead-treated ApoE4-KI females had a significantly lower discrimination index than ApoE4-KI control mice starting at 7 weeks into the lead exposure and this persisted through 10 months post-lead (7 weeks, *p* = 0.0016; 11 weeks, *p* = 0.0002; 3 months, *p* = 0.0030; 10 months, *p* = 0.0269). The lead-treated ApoE4-KI discrimination index was lower than controls at 6 months post-lead but not statistically significant (*p* = 0.0713). **c** Lead-treated ApoE3-KI males had a significantly lower discrimination index at 6 and 10 months post-lead compared to controls (6 months, *p* = 0.0356; 10 months, *p* = 0.0418). **d** In contrast, lead-treated ApoE4-KI males had a significantly lower discrimination index than controls starting at 11 weeks into the lead exposure and this effect persisted through 10 months post-lead exposure (11 weeks, *p* = 0.0064; 4 months, *p* = 0.0433; 6 months, *p* = 0.0382; 10 months, *p* = 0.0275). Data are mean ± SEM with n = 8–13 per sex/genotype/treatment. Multi-level mixed-effects linear regression; significant effect of treatment: # *p* < 0.05; ## *p* < 0.01; ### *p* < 0.001. Two-tailed *t*-test; significant difference between control and lead: n.s., not significant; * *p* < 0.05; ** *p* < 0.01; *** *p* < 0.001

We calculated the discrimination index for each NOL test, and the discrimination indices at each time point are summarized by genotype and sex in Fig. 7. There was an effect of lead exposure on the discrimination index in all lead-exposed animals (Multiple mixed-effects linear regression: ApoE3-KI females, *p* = 0.001; ApoE4-KI females, *p* < 0.0001; ApoE3-KI males, *p* = 0.019; ApoE4-KI males, *p* < 0.0001). During the lead exposure period, there was no difference in the discrimination index between control and lead-treated ApoE3-KI females (Fig. 7a). However, lead-treated ApoE3-KI females had a significantly lower discrimination index at 3 and 10 months post-lead exposure compared to controls.

Interestingly, the lead-treated ApoE4-KI females had a significantly lower discrimination index than control ApoE4-KI females starting at 7 weeks into the lead exposure, and this decrease persisted through 10 months post-lead (Fig. 7b). Thus, lead-treated ApoE3-KI and ApoE4-KI females exhibited persistent deficits in short-term spatial memory, with ApoE4-KI females exhibiting a significant decrease in the discrimination index starting at 7 weeks into the lead exposure, much earlier than lead-treated ApoE3-KI females at 3 months post-lead exposure.

Lead-treated ApoE3-KI males had a significantly lower discrimination index at 6 and 10 months post-lead compared to controls (Fig. 7c), suggesting that spatial memory deficits can develop long after lead exposure. In contrast, lead-treated ApoE4-KI males had a significantly lower discrimination index than controls starting at 11 weeks into the lead exposure and this effect persisted through 10 months post-lead exposure (Fig. 7d). Thus, both ApoE3-KI and ApoE4-KI males exposed to lead exhibited persistent deficits in short-term spatial memory, but the lead-treated ApoE4-KI males showed these deficits much earlier (starting at 11 weeks into the lead exposure vs. 6 months post-lead).

Together, data in Figs. 6 and 7 demonstrate that ApoE4-KI mice exposed to lead developed impaired spatial working memory much earlier than lead-treated ApoE3-KI mice, in both males and females. When we compare males and females of the same genotype, lead-induced spatial memory deficits manifested earlier in females than in males for both the ApoE3-KI and ApoE4-KI mice. These data suggest a GXE between lead exposure and ApoE4 on cognitive impairment as well as sex differences in the onset of cognitive deficits.

### Lead-treated ApoE3-KI and ApoE4-KI mice do not exhibit significant pathology in the DG and other tissues

**Fig. 8:**
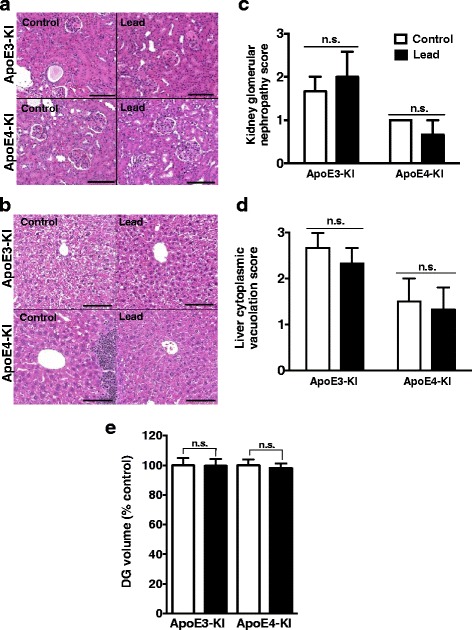
Adult lead does not cause significant liver or kidney toxicity or DG volume loss in aged ApoE3-KI and ApoE4-KI mice. 8-week-old female ApoE3-KI and ApoE4-KI mice were exposed to 0.2% lead acetate for 12 weeks, then switched to normal drinking water and sacrificed 43–45 weeks post-lead exposure (14.5–15 months old). H&E staining of **a** kidney and **b** liver sections and quantification of **c** kidney glomerular nephropathy and **d** liver cytoplasmic vacuolation in female ApoE3-KI and ApoE4-KI mice. **e** DG volume was measured in female ApoE3-KI and ApoE4-KI mice using ImageJ analysis of confocal images of coronal sections from one brain hemisphere. Data are mean ± SEM. *n* = 3–5 per genotype/treatment. Two-way ANOVA with Fisher’s LSD *post-test*: n.s., not significant. Scale bars, 100 μm

**Fig. 9:**
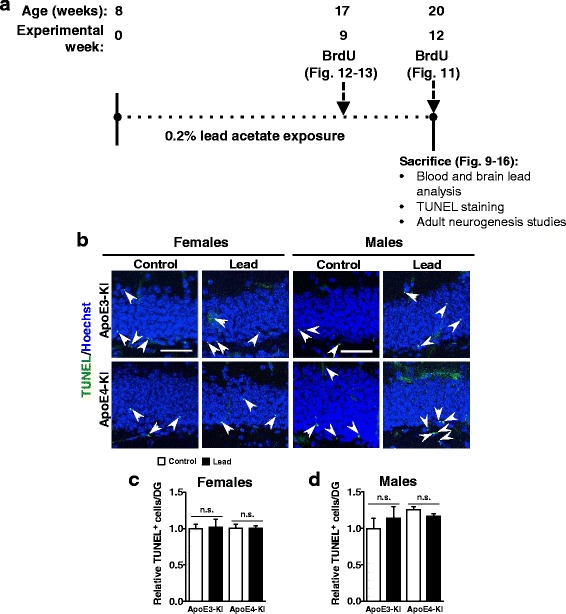
Adult-only lead exposure does not increase the number of TUNEL^+^ apoptotic cells in the DG of the hippocampus. **a** Experimental design and timeline for cellular studies (BrdU dosing) for Figs. 9, 10, 11, 12, 13, 14, 15 and 16. **b** Representative images of TUNEL^+^ (green) cells in the DG of control and lead-treated ApoE3-KI and ApoE4-KI males and females. Quantification of TUNEL^+^ cells per DG in ApoE3-KI and ApoE4-KI **c** females and **d** males*.* Data are mean ± SEM with *n* = 3–4 per genotype/sex/treatment. Two-way ANOVA with Fisher’s LSD *post-test*: n.s., not significant. Scale bar, 50 μm

In order to determine if lead exposure causes systemic toxicity, we assessed for pathological changes in the liver and kidney of 64-week-old (~15-month-old) male and female ApoE3-KI and ApoE4-KI mice after various behavioral testing. There were no significant differences in kidney or liver pathology scores between control and lead-treated mice of either sex or genotype (Fig. 8a-d and data not shown). Furthermore, there were no changes in the volume of the DG of the hippocampus in ApoE3-KI or ApoE4-KI females upon lead exposure (Fig. 8e). In addition, we did not observe an increase in TUNEL^+^ cells in the hippocampus or cortex of lead-treated animals (Fig. 9). Thus, the lead exposure regimen used in this study did not result in gross pathological changes in the DG, kidney, or liver.

### Adult lead exposure results in elevated blood lead levels and lead deposition in the brain

**Fig. 10:**
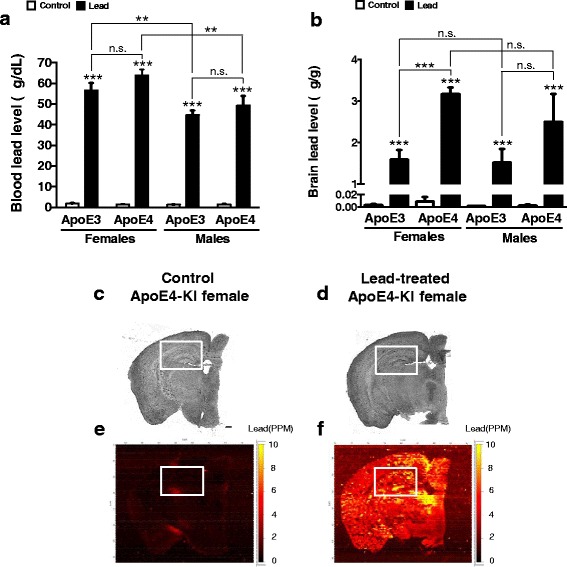
Adult-only lead exposure results in elevated blood lead levels and lead deposition in the brain. 8-week-old ApoE3-KI and ApoE4-KI male and female mice were exposed to 0.2% lead acetate for 12 weeks and then sacrificed. Blood and one brain hemisphere were collected at sacrifice and (**a**) blood lead and (**b**) brain lead levels were measured using ICP-MS. Brightfield images of one brain hemisphere from a female (**c**) control and (**d**) lead-treated ApoE4-KI mouse after the 12 week lead exposure. Semi-quantitative measurement of lead in the brain of a (**e**) control and (**f**) lead-treated ApoE4-KI mouse using LA-ICP-MS. Two-way ANOVA with Fisher’s LSD *post-test*: n.s., not significant; ** *p* < 0.01; *** *p* < 0.001. Scale bars, 100 μm

We collected blood and brain tissue for lead analysis from the cellular cohort at sacrifice after the 12-week lead exposure. Lead exposure significantly raised blood lead levels in all animals relative to controls at the end of the 12 week exposure, and lead levels were higher in exposed females than males of the same genotype (Fig. 10a). Lead-exposed animals also had significantly higher brain lead levels relative to their controls at the end of the 12-week exposure, with the highest levels in ApoE4-KI female brains (Fig. 10b). Low-resolution LA-ICP-MS of sections from a control and a lead-treated female ApoE4-KI mouse illustrate that adult-only lead exposure is sufficient to cause increased lead deposition throughout the brain (Fig. 10c-f), while levels of the essential metal zinc (an internal control for the imaging and exposure) were not significantly different between control and lead-treated mice (data not shown).

### Lead decreases proliferation of adult-born cells in the hippocampus in all lead-exposed mice

Adult hippocampal neurogenesis is the process whereby adult neural stem cells in the DG of the hippocampus generate adult-born neurons [31]. These adult-born neurons can influence hippocampus-dependent learning and memory [33, 34, 37, 55]. Interestingly, ApoE4 alters adult-born neuron differentiation and maturation in the DG of female ApoE4-KI mice [40]. Thus, we examined whether adult lead exposure inhibits adult neurogenesis in the hippocampus and whether there is a GXE between lead exposure and ApoE4 on this process using a second cohort (the cellular cohort) of mice.

**Fig. 11:**
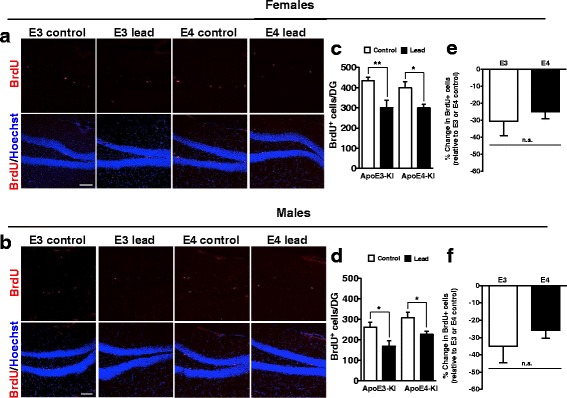
Adult lead exposure decreases adult-born cell proliferation in the DG of the hippocampus of all mice. 8-week-old male and female ApoE3- and ApoE4-KI mice were exposed to 0.2% lead acetate for 12 weeks and then sacrificed. BrdU was administered 2 h prior to sacrifice (1 x 100 mg/kg). Representative images of BrdU (red) immunostaining in the DG of 20-week-old (**a**) female and (**b**) male ApoE3-KI and ApoE4-KI control and lead-treated mice. Quantification of the total BrdU+ cells per DG in (**c**) females and (**d**) males. Quantification of the percent change in total BrdU+ cells in lead-treated mice relative to ApoE3-KI or ApoE4-KI control (**e**) females and (**f**) males. Data are mean ± SEM with *n* = 3–5 per genotype/sex/treatment. Two-way ANOVA with Fisher’s LSD *post-test*: n.s., not significant; * *p* < 0.05; ** *p* < 0.01. Scale bars, 100 μm

To determine if adult-only lead exposure is sufficient to impair cell proliferation during adult neurogenesis, we treated half of the cellular cohort of mice with BrdU 2 h prior to sacrifice at the end of the 12-week lead exposure in order to label actively proliferating cells in S-phase of the cell cycle (Fig. 11). We found that lead significantly reduced the total number of BrdU^+^ cells in all lead-treated animals compared to their respective controls of the same genotype and sex (Fig. 11a-d). The percent change in BrdU^+^ cells in lead-treated mice compared to control mice was not statistically significantly different between ApoE3-KI and ApoE4-KI mice, in either sex (Fig. 11e-f). These data suggest that adult-only lead exposure for 12 weeks is sufficient to significantly decrease adult-born cell proliferation in the DG of the hippocampus of all mice. Although this cellular mechanism does not explain the behavior differences observed between ApoE3-KI vs. ApoE4-KI mice, or between males and females, it may contribute to the eventual memory loss in all lead-exposed mice, such as that observed in the novel object location test.

### Lead impairs adult-born neuron differentiation and decreases the dendritic complexity of immature neurons in the DG of female ApoE4-KI mice

**Fig. 12:**
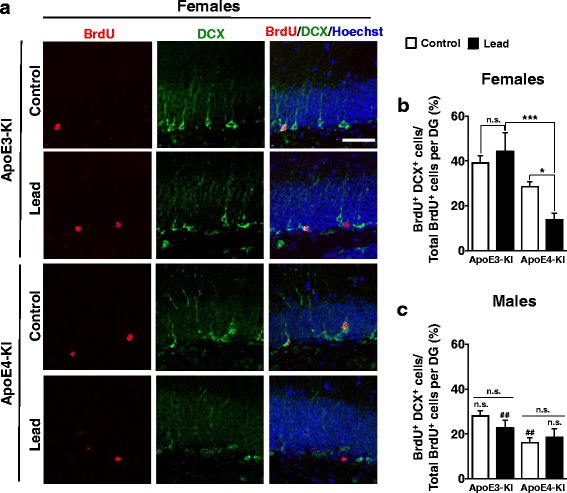
Lead decreases adult-born immature neuron maturation in the DG of ApoE4-KI female mice. 8-week-old male and female ApoE3- and ApoE4-KI mice were exposed to 0.2% lead acetate for 12 weeks and then sacrificed. 100 mg/kg BrdU was administered 5 times in 1 day (every 2 h) 3 weeks prior to sacrifice (i.e., at 9 weeks into lead exposure). **a** Representative images of BrdU (red) and DCX (green) immunostaining in the DG of control and lead-treated female ApoE3-KI and ApoE4-KI animals. Quantification of the percent of total BrdU^+^ cells that are BrdU^+^DCX^+^ per DG in females (**b**) and males (**c**) respectively. Data are mean ± SEM with *n* = 4–5 per genotype/sex/treatment. Two-way ANOVA with Fisher’s LSD *post-test*: Comparison between control and lead-treated: n.s., not significant; * *p* < 0.05; *** *p* < 0.001. Comparison between males and females of the same genotype and treatment: n.s., not significant; ##, *p* < 0.01. Scale bar, 50 μm

**Fig. 13:**
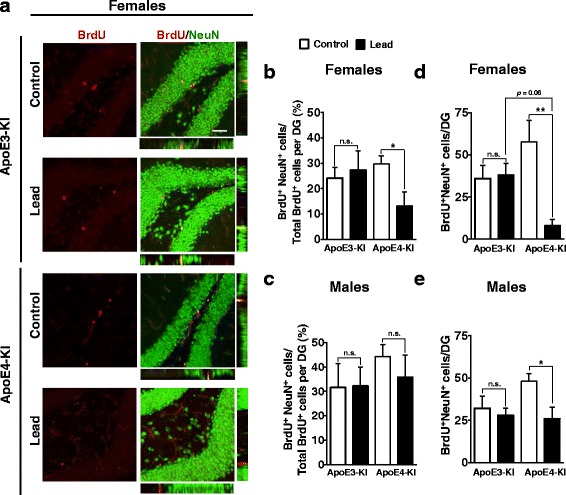
Lead decreases adult-born neuron differentiation in the DG of ApoE4-KI female mice. 8-week-old male and female ApoE3-KI and ApoE4-KI mice were exposed to 0.2% lead acetate for 12 weeks and then sacrificed. 100 mg/kg BrdU was administered 5 times in 1 day (every 2 h) 3 weeks prior to sacrifice (i.e., at 9 weeks into lead exposure). **a** Representative images of BrdU (red) and NeuN (green) immunostaining in the DG of control and lead-treated female ApoE3-KI and ApoE4-KI animals. Quantification of the percent of total BrdU+ cells that are BrdU + NeuN+ per DG in **b** females and **c** males, respectively. Quantification of the total number of BrdU + NeuN+ cells in **d** females and **e** males, respectively. Data are mean ± SEM with *n* = 4–5 per genotype/sex/treatment. Two-way ANOVA with Fisher’s LSD *post-test*: n.s., not significant; * *p* < 0.05; ** *p* < 0.01. Scale bar, 50 μm

We examined the effect of lead on neuronal differentiation of adult-born cells in the DG, using immunostaining for DCX and NeuN as markers of immature and mature neurons, respectively (Figs. 12a and 13a). BrdU was administered to the remaining half of the cellular cohort of mice at 9 weeks into the lead exposure (3 weeks prior to sacrifice) in order to label 3-week-old adult-born cells. Lead treatment significantly reduced the proportion of adult-born cells that express DCX (BrdU^+^DCX^+^/total BrdU^+^) or NeuN (BrdU^+^NeuN^+^/total BrdU^+^) in female ApoE4-KI mice, but not in other lead-treated mice (Fig. 12b-c, and Fig. 13b-c), suggesting that lead inhibits neuronal differentiation of adult-born neurons in the hippocampus and that female ApoE4-KI mice are more sensitive. There was also a statistically significant decrease in the number of total adult-born mature neurons (BrdU^+^NeuN^+^) in both male and female ApoE4-KI mice, although the effect was smaller in males than in females (Fig. 13d-e).

**Fig. 14:**
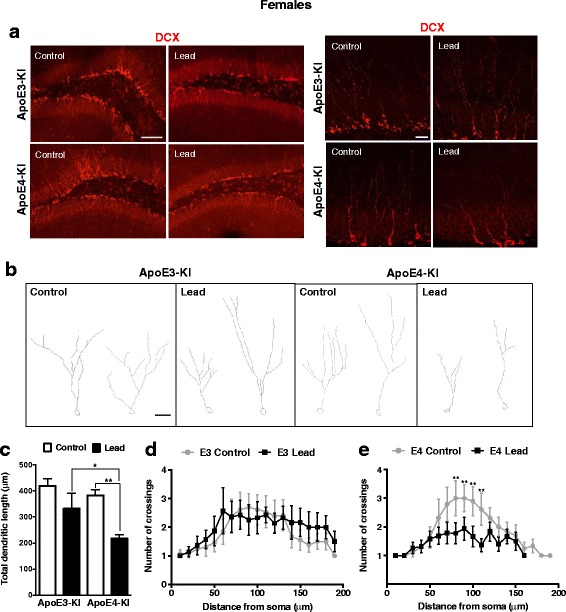
Lead decreases the dendritic complexity of immature neurons in the DG of ApoE4-KI female mice. **a**, Representative confocal images of DCX (red) immunostaining in the DG of 20-week-old female ApoE3-KI and ApoE4-KI control and lead-treated mice (left scale bar, 100 μm; right scale bar, 25 μm). **b**, Representative examples of DCX+ neurons from female ApoE3-KI and ApoE4-KI control and lead-treated mice traced using the ImageJ Simple Neurite Tracer plug-in (scale bar, 25 μm). **c**, Quantification of the total dendritic length of DCX+ neurons in the DG. Sholl analysis of DCX+ neurons from (**d**) ApoE3-KI and (**e**) ApoE4-KI female mice. Data are mean ± SEM with *n* = 4–5 per genotype/treatment. Two-way ANOVA with Fisher’s LSD *post-test* for analysis of total dendritic length; two-tailed *t-test* for within genotype comparisons of the number of crossings in control vs. lead-treated mice: * *p* < 0.05; ** *p* < 0.01

**Fig. 15:**
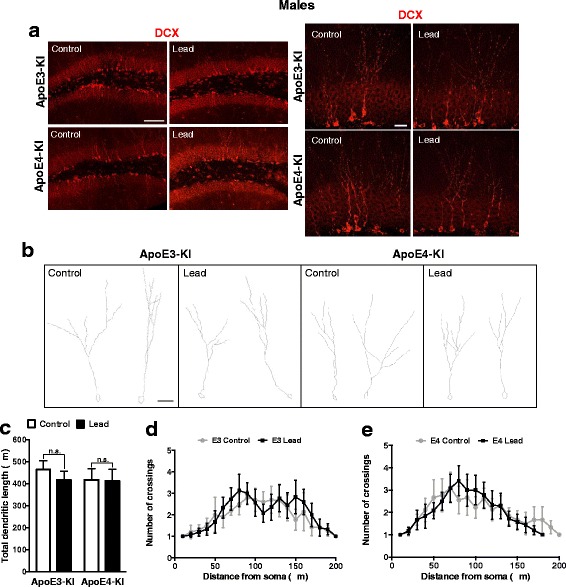
Lead does not impair the dendritic complexity of immature neurons in the DG of male mice. **a**, Representative confocal images of DCX (red) immunostaining in the DG of 20-week-old male ApoE3-KI and ApoE4-KI control and lead-treated mice (left scale bar, 100 μm; right scale bar, 25 μm). **b**, Representative examples of DCX+ neurons from male ApoE3-KI and ApoE4-KI control and lead-treated mice traced using the ImageJ Simple Neurite Tracer plug-in (scale bar, 25 μm). **c**, Quantification of the total dendritic length of DCX+ neurons in the DG. Sholl analysis of DCX+ neurons from **d** ApoE3-KI and **e** ApoE4-KI male mice. Data are mean ± SEM with *n* = 4–5 per genotype/treatment. Two-way ANOVA with Fisher’s LSD *post-test*: n.s., not significant

We also assessed the dendritic morphology, a measure of neuronal maturation, of adult-born neurons in this same cohort of mice. Because the acid treatment necessary for BrdU staining damages neuronal processes, we used DCX immunostaining alone to visualize neuronal processes of newly generated neurons (Fig. 14a-b, 15a-b). Neurite tracing and Sholl analysis showed that lead treatment significantly decreased the total dendritic length (Fig. 14c) and the number of dendritic crossings (Fig. 14e) in female ApoE4-KI mice. This effect was not seen in female ApoE3-KI mice (Fig. 14d) or any of the male mice (Fig. 15). These data suggest that ApoE4-KI females are more sensitive to the effects of lead on neuronal differentiation and maturation.

### Lead reduces the number of GABAergic interneurons in the DG of ApoE4-KI female mice

**Fig. 16:**
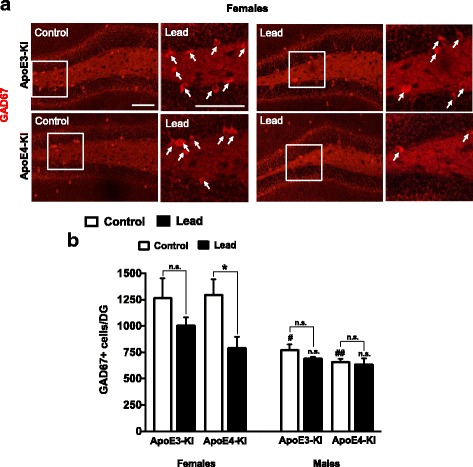
Lead decreases the total number of GAD67+ GABAergic interneurons in 20-week-old female ApoE4-KI mice. 8-week-old male and female ApoE3-KI and ApoE4-KI mice were exposed to 0.2% lead acetate for 12 weeks and sacrificed. **a** Representative images of GAD67 (red) immunostaining in the DG of 20-week-old female ApoE3-KI and ApoE4-KI control and lead-treated mice. **b** Quantification of the total number of GAD67+ cells in the DG of 20-week-old ApoE3-KI and ApoE4-KI females and males. Data are mean ± SEM with *n* = 3–5 per genotype/treatment. Two-way ANOVA with Fisher’s LSD *post-test*. Comparison between control and lead-treated: n.s., not significant; * *p* < 0.05. Comparison between males and females of the same genotype and treatment: n.s., not significant; #, *p* < 0.05; ##, *p* < 0.01. Scale bars, 100 μm

GABAergic interneurons play an important role in adult hippocampal neurogenesis [56]. Previous studies report that ApoE4-KI female mice exhibit an age-dependent decrease in the number of GABAergic interneurons in the DG of the hippocampus, starting at 6 months of age [12, 14, 40]. Thus, we examined the effect of lead on the number of GABAergic neurons at the end of the 12-week lead exposure (20-weeks old) using GAD67 staining as a marker (Fig. 16a). There was no significant difference in the total number of GABAergic neurons between control ApoE3-KI and ApoE4-KI mice in either sex (Fig. 16b). However, lead treatment significantly decreased the number of GAD67^+^ cells in the DG of ApoE4-KI females but not in other mice.

## Discussion

AD currently affects nearly 35 million people and is projected to affect 117 million people worldwide by 2050 [57]. AD and cognitive decline are associated with significant societal, financial, and health care costs. Yet, there is a paucity of research on the role of environmental exposures and GXE on neurodegenerative disease risk and AD pathogenesis. Thus, additional investigation into how environmental factors and GXE may influence AD risk or accelerate cognitive decline is of immense public health importance.

Functional impairment in cognition is often used as a proxy to estimate the presence and degree of AD-associated neuropathology [58, 59]. Here, we used humanized transgenic knock-in mice as an animal model of human ApoE4 carriers to assess whether there is a GXE between lead and ApoE4 on cognitive behavior, and potentially on AD risk. ApoE3-KI and ApoE4-KI female mice develop spontaneous learning and memory deficits starting at 15–18 months [12–14]. Consequently, we assessed learning and memory prior to 15 months of age to determine if lead can accelerate the onset of cognitive behavior deficits. We defined a GXE as a different effect of lead exposure on cognitive behavior in mice expressing the human ApoE4 allele from those expressing the human ApoE3 allele. Based on this definition, we concluded that lead interacts with ApoE4 because we observed statistically significant deficits in contextual fear memory and spontaneous alternation in lead-treated ApoE4-KI female mice but not in other animals. In addition, lead causes an earlier onset of spatial working memory deficits in the NOL test in lead-treated ApoE4-KI mice compared to ApoE3-KI mice, for both males and females. Together, these data suggest that the effect of lead exposure on cognitive behavior is different depending on ApoE genotype. More specifically, a GXE between lead and ApoE4 may accelerate cognitive decline, contribute to a more rapid progression from cognitive aging to mild cognitive impairment, and potentially contribute to AD. Furthermore, females may be more susceptible to this GXE.

We found that lead exposure decreased spontaneous alternation in the T-maze in female ApoE4-KI animals. While alternation is not strictly hippocampus-dependent, alternation performance is very sensitive to hippocampal lesions [44, 54, 60]. More importantly, impaired alternation and/or repetitive arm entry has been reported in several different AD-mouse models, including models with presenilin and amyloid precursor protein mutations [61–64].

Lead exposure also impaired hippocampus-dependent spatial memory in the NOL test in all lead-treated animals. Interestingly, lead-exposed ApoE3-KI male mice developed memory loss in the NOL test at 6 months post-lead exposure. Moreover, the memory loss in all lead-exposed animals was persistent through 10 months post-lead. These data suggest that sub-chronic adult lead exposure alone is sufficient to cause long lasting or irreversible memory impairment, and that this behavior phenotype can occur long after exposure has ended. These findings are pertinent to human health because irreversible loss of memory formation is characteristic of AD, senile dementia, and up to 50% of Parkinson’s disease cases [65]. Importantly, the aging population in the U.S. experienced high lead exposure in early life [66]. Thus, it is possible that lead exposure may lead to cognitive impairment later in life and contribute to cognitive decline associated with aging and neurodegeneration, such as late-onset AD.

Epidemiological reports suggest an increased risk of late-onset AD and increased cognitive decline with mild cognitive impairment in women [15, 16]. We observed that lead impaired contextual fear memory and working memory in the T-maze in female but not male ApoE4-KI mice. Furthermore, lead-induced spatial memory impairment in the NOL test manifested earlier in females than males for both ApoE3-KI and ApoE4-KI mice. These data provide strong evidence for sex differences in susceptibility to lead-induced cognitive decline in an animal model and suggest that women may be more susceptible to cognitive impairment and/or AD following exposure to toxicants such as lead.

We report average peak blood lead levels of 44.4–63.4 μg/dL, similar to previous animal studies and comparable to some occupational exposure settings in the 20th century [49, 67–69]. Elevated blood lead levels (≥10 μg/dL for adults) can still be encountered among U.S. workers, and Occupational Safety & Health Administration regulations do not require U.S. workers to be removed from their job sites until they have a blood lead level ≥ 50 μg/dL. [70]. Importantly, high levels of lead exposure from environmental sources and in occupational settings are still a major concern in developing countries [18, 71]. Therefore, 0.2% lead acetate serves as an experimental model for occupational exposures globally, for environmental exposure in developing countries, and for the US population who were exposed to high levels of environmental lead prior to the ban of leaded gasoline and paint.

Brain and blood lead levels were highest in female ApoE4-KI mice. There were no differences in water consumption during the first week but water consumption thereafter was not monitored. Although we cannot formally exclude the possibility that ApoE4-KI females drank more water than other animals after the first week of lead exposure, there was no statistically significant difference between blood lead levels of female ApoE3-KI and ApoE4-KI mice. Thus, it seems unlikely that higher water consumption alone led to more lead deposition in the brain of female ApoE4-KI.

It is possible that the ApoE4-KI enhances lead deposition in the brain and differences in brain lead disposition may contribute to the GXE between ApoE4 and lead. Interestingly, ApoE4-KI mice exhibit decreased basement membrane thickness, reduced collagen and tight junction protein expression, as well as increased matrix metalloproteinase 9 activity in the blood-brain barrier, suggesting that more lead may accumulate in the brain of ApoE4-KI mice due to reduced blood-brain barrier integrity [72–74]. Furthermore, human ApoE4 carriers with AD also exhibit a thinner basement membrane, increased fibrin extravasion, and pericyte loss in the blood-brain barrier compared to ApoE3-KI carriers with AD or non-AD controls [75, 76]. Thus, effects of E4 on the blood-brain barrier may increase the accumulation of lead in the brain in ApoE4-KI mice or maybe even in humans, and contribute to more severe or earlier onset of learning and memory impairments.

We previously found that lead can impair the survival, proliferation, and differentiation of adult neural precursor cells in vitro [41]. Thus, we sought to determine whether lead can interact with ApoE4 to impair adult hippocampal neurogenesis in vivo and whether this could be an underlying mechanism for the observed cognitive deficits. We exposed mice to lead starting at 8 weeks of age in order to avoid the cumulative effects of lead on both the developing and adult brain [77]. Unlike previous reports using the ApoE3-KI and ApoE4-KI models, we did not see a significant effect of ApoE4 alone on adult-born cell proliferation and maturation. This may be due to the fact that the effects of ApoE4 are age-dependent and we analyzed adult neurogenesis 1.5–2.5 months earlier (in 4.6-month-old vs. 6–7-month-old mice) than previous studies [12, 14, 40]. Data presented here demonstrate that adult lead exposure impairs adult-born cell proliferation in all lead-treated animals; this mechanism may contribute to lead impairment of spatial memory in the novel object location test that eventually develop in all lead-treated mice. Furthermore, lead exposure significantly impaired adult-born neuron differentiation and decreased dendritic complexity specifically in ApoE4-KI females. These cellular changes correlate with the fact that lead impairment of cognition is most pronounced in ApoE4-KI females. There was also a statistically significant reduction in the number of adult-born mature neurons (BrdU^+^NeuN^+^ cells) in male ApoE4-KI mice, although the reduction is smaller than that in females. This cellular change occurs at the end of the 12-week lead exposure and correlates with the onset of NOL deficits in the lead-treated ApoE4-KI males in the behavior cohort. Together, these data suggest that lead and ApoE4 may interact to perturb neuronal differentiation and maturation during adult hippocampal neurogenesis, and that this mechanism may contribute to the lead and ApoE4 interaction on accelerated cognitive impairment in ApoE4 mice and the heightened sensitivity of ApoE4 females.

The survival and integration of adult-born neurons is activity-dependent and occurs at approximately three weeks after neuronal birth [78, 79]. Several different cell types and inputs facilitate the survival and maturation of adult-born cells during this window, including GABAergic interneurons that may exert trophic effects on adult-born immature neuron survival and maturation [56, 80, 81]. Previous studies found that there are basal sex differences in the number of GAD67^+^ interneurons in ApoE3-KI and ApoE4-KI mice, with female mice having significantly more GAD67^+^ interneurons, and that female ApoE4-KI mice have significantly fewer GAD67^+^ cells than ApoE3-KI mice starting at 6–7 months of age [40]. We found that lead significantly decreased the total number of GAD67^+^ cells in 20-week-old, lead-treated ApoE4-KI females. This cellular effect correlates closely with our observation of significantly reduced adult-born neuron maturation and dendritic complexity in ApoE4-KI females, suggesting that an interaction between lead and ApoE4 may contribute to earlier impairment of GABAergic input and contribute to the observed impairment in immature neuron maturation in ApoE4-KI females.

Adult-born, immature neurons play a critical role in synaptic plasticity within the DG [79, 82, 83]. Notably, the increased responsiveness and excitability of newborn granule cells may give these adult-born cells a specific and significant role in learning and memory [55, 84]. In rats, a greater proportion of young, adult-born neurons are activated during spatial exploration compared to existing granule cells [85, 86]. Similarly, the selective loss of immature neurons impairs long-term spatial memory retention and contextual fear extinction [36]. Thus, the disruption of adult-born immature neuron maturation in lead-treated ApoE4-KI females may be associated with the impaired hippocampus-dependent learning and memory in these mice.

Although lead was deposited in the mouse brain broadly including the hippocampus and no regional specific deposition was found, we did not observe overt motor impairment. In humans, high levels of lead exposure can cause fine and gross motor impairments. However, lead exposure during development, which interferes with the proper development of the nervous system, likely plays a significant role in these cases. In the present study, mice were exposed to lead beginning at 8 weeks of age, eliminating the effect of lead on the developing nervous system. We only measured gross motor function (open field test), so we cannot rule out if lead exposure caused fine motor dysfunction. We did not observe a significant increase in TUNEL^+^ cells in the hippocampus or cortex of lead-treated animals. However, lead impaired adult neurogenesis in the hippocampus, with the greatest effect seen in female ApoE4-KI mice. Thus, newborn cells and neurons in the hippocampus, especially those in mice expressing human ApoE4 allele, may be more vulnerable than neurons in other regions of the brain to lead toxicity in the adult mice.

Similar to other studies that have reported basal sex differences in adult neurogenesis in these mice [14], we report baseline differences in the total number of adult-born cells (total BrdU^+^ cells), and the total number of GAD67^+^ cells in the DG between control males and females. However, we do not think these differences alter the conclusions of this study. For example, we found that all control mice performed equally well in tests for contextual fear memory, spontaneous alternation in the T-maze, and memory for novel object location (NOL). In addition, while ApoE3-KI and ApoE4-KI male mice had a lower number of BrdU^+^ and GAD67^+^ cells, they did not show statistically significant memory deficits after lead exposure when tested for contextual fear memory or spontaneous alternation in the T-maze. They also exhibited a later onset of deficits in the NOL test than their respective females of the same genotype. These data suggest that the lower basal adult neurogenesis in males does not affect their basal shot-term spatial and contextual memory, nor lead to higher sensitivity to lead impairment of cognition.

## Conclusions

Although it has been hypothesized that exposure to environmental factors and a GXE interaction may increase AD risk, there is little direct evidence supporting this hypothesis. In this study, we found that adult lead exposure is sufficient to impair hippocampus-dependent learning and memory and that these deficits are more severe or occur earlier in female ApoE4-KI mice. These findings provide some of the first direct evidence for a GXE interaction between lead and ApoE4 on cognitive impairment. Furthermore, impaired adult hippocampal neurogenesis may be a potential mechanism underlying this GXE interaction. These results also provide new insight into how environmental toxicants and genetic risk factors may interact to contribute to cognitive decline and neurodegeneration in non-AD patients.

## Abbreviations

AD: Alzheimer’s disease
ApoE3-KI and ApoE4-KI: Apolipoprotein E3 or E4 knock-in mouse model
BrdU: 5-bromo-2′-deoxyuridine
DG: Dentate gyrus
GXE: Gene-environment interaction
H&E: Hematoxylin and eosin
ICP-MS: Inductively coupled mass spectrometry
NOL: Novel Object Location
