# Correction to: Gene-environment interaction between lead and Apolipoprotein E4 causes cognitive behavior deficits in mice

**DOI:** 10.1186/s13024-017-0223-7

**Published:** 2017-11-03

**Authors:** Anna K. Engstrom, Jessica M. Snyder, Nobuyo Maeda, Zhengui Xia

**Affiliations:** 1 Toxicology Program, Department of Environmental and Occupational Health Sciences, University of Washington, Box 357234, Seattle, WA 98195 USA; 2 Department of Comparative Medicine, School of Medicine, University of Washington, Seattle, WA 98195 USA; 3 Department of Pathology and Laboratory Medicine, University of North Carolina, Chapel Hill, NC 27599 USA

## Erratum

The original article [1] contains an error in the y-axes of Fig. 10a & b – the accidental omission of a μ symbol preceding the denoted units for both graphs means that incorrect units are displayed.

**Fig. 10:**
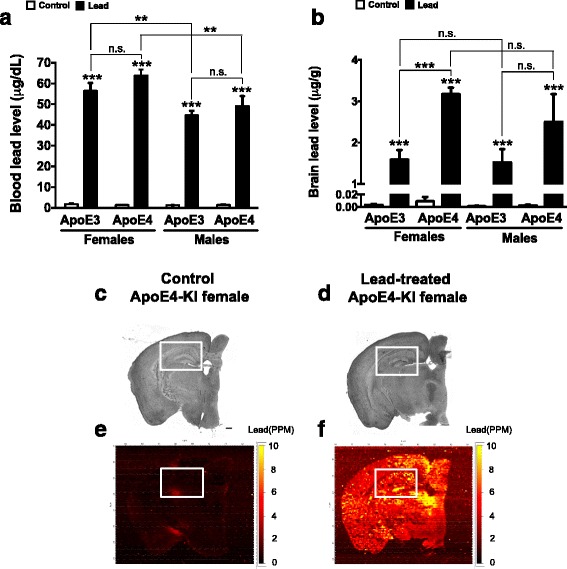
Adult-only lead exposure results in elevated blood lead levels and lead deposition in the brain. 8-week-old ApoE3- KI and ApoE4-KI male and female mice were exposed to 0.2% lead acetate for 12 weeks and then sacrificed. Blood and one brain hemisphere were collected at sacrifice and (**a**) blood lead and (**b**) brain lead levels were measured using ICPMS. Brightfield images of one brain hemisphere from a female (**c**) control and (**d**) lead-treated ApoE4-KI mouse after the 12 week lead exposure. Semi-quantitative measurement of lead in the brain of a (**e**) control and (**f**) lead-treated ApoE4-KI mouse using LA-ICP-MS. Two-way ANOVA with Fisher’s LSD post-test: n.s., not significant; ** *p* < 0.01; *** *p* < 0.001. Scale bars, 100 μm

As such, the authors would like to note that the correct units are μg/dL for Fig. 10a and μg/g for Fig. 10b.

The correct version of this figure is displayed below for reference too.
